# eHealth Literacy and Web-Based Health Information–Seeking Behaviors on COVID-19 in Japan: Internet-Based Mixed Methods Study

**DOI:** 10.2196/57842

**Published:** 2024-07-11

**Authors:** Seigo Mitsutake, Koichiro Oka, Orkan Okan, Kevin Dadaczynski, Tatsuro Ishizaki, Takeo Nakayama, Yoshimitsu Takahashi

**Affiliations:** 1 Human Care Research Team Tokyo Metropolitan Institute for Geriatrics and Gerontology Tokyo Japan; 2 Australian Institute of Health Innovation Macquarie University NSW Australia; 3 Faculty of Sport Sciences Waseda University Saitama Japan; 4 WHO Collaborating Centre for Health Literacy, Department of Health and Sport Sciences TUM School of Medicine and Health Technical University of Munich Munich Germany; 5 Department of Health Science Fulda University of Applied Science Fulda Germany; 6 Center for Applied Health Science Leuphana University of Lueneburg Lueneburg Germany; 7 Human Care Research Team Tokyo Metropolitan Institute for Geriatrics and Gerontology Itabashi-ku Japan; 8 Department of Health Informatics Kyoto University School of Public Health Kyoto Japan

**Keywords:** COVID-19, infectious, public health, SARS-COV-2, respiratory, eHealth, health communication, web-based information, DHLI, eHEALS, internet, mixed methods study, adult population, Asia, Asian, cross sectional, survey, surveys, questionnaire, questionnaires, Japan, Japanese, information seeking, information behavior, information behavior, health literacy, eHealth literacy, digital health literacy

## Abstract

**Background:**

During the COVID-19 pandemic, much misinformation and disinformation emerged and spread rapidly via the internet, posing a severe public health challenge. While the need for eHealth literacy (eHL) has been emphasized, few studies have compared the difficulties involved in seeking and using COVID-19 information between adult internet users with low or high eHL.

**Objective:**

This study examines the association between eHL and web-based health information–seeking behaviors among adult Japanese internet users. Moreover, this study qualitatively shed light on the difficulties encountered in seeking and using this information and examined its relationship with eHL.

**Methods:**

This cross-sectional internet-based survey (October 2021) collected data from 6000 adult internet users who were equally divided into sample groups by gender, age, and income. We used the Japanese version of the eHL Scale (eHEALS). We also used a Digital Health Literacy Instrument (DHLI) adapted to the COVID-19 pandemic to assess eHL after we translated it to Japanese. Web-based health information–seeking behaviors were assessed by using a 10-item list of web sources and evaluating 10 topics participants searched for regarding COVID-19. Sociodemographic and other factors (eg, health-related behavior) were selected as covariates. Furthermore, we qualitatively explored the difficulties in information seeking and using. The descriptive contents of the responses regarding difficulties in seeking and using COVID-19 information were analyzed using an inductive qualitative content analysis approach.

**Results:**

Participants with high eHEALS and DHLI scores on information searching, adding self-generated information, evaluating reliability, determining relevance, and operational skills were more likely to use all web sources of information about COVID-19 than those with low scores. However, there were negative associations between navigation skills and privacy protection scores when using several information sources, such as YouTube (Google LLC), to search for COVID-19 information. While half of the participants reported no difficulty seeking and using COVID-19 information, participants who reported any difficulties, including *information discernment*, *incomprehensible information*, *information overload*, and *disinformation*, had lower DHLI score. Participants expressed significant concerns regarding “information quality and credibility,” “abundance and shortage of relevant information,” “public trust and skepticism,” and “credibility of COVID-19–related information.” Additionally, they disclosed more specific concerns, including “privacy and security concerns,” “information retrieval challenges,” “anxieties and panic,” and “movement restriction.”

**Conclusions:**

Although Japanese internet users with higher eHEALS and total DHLI scores were more actively using various web sources for COVID-19 information, those with high navigation skills and privacy protection used web-based information about COVID-19 cautiously compared with those with lower proficiency. The study also highlighted an increased need for information discernment when using social networking sites in the “Health 2.0” era. The identified categories and themes from the qualitative content analysis, such as “information quality and credibility,” suggest a framework for addressing the myriad challenges anticipated in future infodemics.

## Introduction

### Background

The internet is a powerful source of information on health behavior, health, and medical care. Most of the general adult population uses the internet in Japan, as in other high-income countries [1-3]. Approximately 73% of Japanese internet users have searched for health information in the past 12 months [4]. However, many websites providing health information are unreliable and may be more linked to promoting commercial goods or private health services [5-7]. Misinformation (ie, false information distributed without the intention to cause harm) and disinformation (ie, false information shared deliberately to cause harm) may negatively affect people’s physical and mental health, increase stigmatization, and threaten precious health gains, which lead to poor observance of public health measures [8,9]. Therefore, eHealth literacy (eHL), defined as the ability to seek, find, understand, and appraise health information on the internet to address or solve a health problem, is essential for accessing and using reliable health information via the internet.

During the COVID-19 pandemic, an “infodemic”—an epidemic of misinformation or disinformation—emerged and spread rapidly via the internet, posing a severe public health problem [10]. The COVID-19 infodemic has highlighted that poor eHL is a major challenge in using COVID-19 information on the internet [11]. People with poor health literacy are more likely to be confused by COVID-19 information on the internet [12]. Therefore, there is a need to improve health communication strategies for people with poor eHL to access reliable COVID-19 information on the internet easily.

Understanding the COVID-19 information–seeking behavior and identifying the difficulties internet users with low eHL are confronted with when dealing with this information are essential for improving communication strategies on COVID-19 and other health crises. The COVID-19 health literacy (COVID-HL) network surveyed digital health literacy (DHL), defined to have the same meaning as eHL [13]. Studies of the COVID-HL network revealed that university students with low eHL were more likely to use social media but less likely to use search engines and websites of official institutions than those with high eHL using quantitative data [14-18]. However, few studies have compared the difficulties individuals encounter when seeking and using COVID-19 information identified by qualitative content analysis between internet users with low and high eHL as estimated by an assessment tool. Mixed methods analyses, which integrate both quantitative and qualitative data, could increase our understanding of these difficulties and inform the development of strategies to enhance eHL for all individuals and improve the quality of web-based content. In addition, a limitation of these prior studies was that they included only college students [14-18] or physicians [19]. Examining the associations of eHL with health information–seeking behavior among other age groups is needed because the internet is used by not only younger adults but also different age groups, and older adults are reported to have barriers to seeking health information from the internet [1,2].

### Objective

Comparing the subjective difficulties in seeking information between internet users with high and low eHL would help improve the strategies promoting access to reliable COVID-19 information. Therefore, this study aimed to examine the association between eHL and web-based health information–seeking behaviors using a mixed methods strategy. In addition, this study aimed to qualitatively shed light on the difficulties encountered in seeking and using this information, and to examine its relationship with eHL.

## Methods

### Study Design and Setting

This study used data from a cross-sectional internet-based survey that was conducted in Japan in October 2021. The study participants were recruited from the registrants of a Japanese internet research company (MyVoice Communication, Inc), who were asked to respond to the survey. This research company has approximately 553,719 registrants who could respond to this survey and obtained detailed sociodemographic data from each participant upon registration in 2021.

### Study Participants

This study aimed to collect data from 6000 men and women aged 20 to 79 years. The participants were equally divided into 132 sample groups categorized by gender (men and women), age (6 categories: 20-29, 30-39, 40-49, 50-59, 60-69, and 70-79 years), and income (11 categories: <1, 1-<2, 2-<3, 3-<4, 4-<5, 5-<6, 6-<7, 7-<8, 8-<9, 9-<10, ≥10 million Yen [1 Yen=US $0.0088]; October 2021), with 45 participants in each group. The internet research service company randomly chose 250 potential respondents to include 45 participants in each group from the registered participants in accordance with the company’s response rate data. Potential respondents could log into a protected site area using a unique ID and password. After the desired number of participants voluntarily signed a web-based informed consent form and completed a sociodemographic information form, further participants were no longer accepted.

### Ethical Considerations

The Ethics Committees of the Tokyo Metropolitan Institute for Geriatrics and Gerontology (R21-055) and Kyoto University (R3191) approved the study protocol. All procedures followed the Ethical Guidelines of the Medical and Biological Research Involving Human Subjects established by the Japanese government. Data for analysis was provided by the research company after deidentification. Finally, we obtained informed consent from participants before the survey. Reward points valued at 130 Yen were provided as incentives for participation.

### Measures

#### Exposure: eHL

The Japanese version of the eHL Scale (J-eHEALS) was used to assess eHL for using health information on the internet as a 1-way communication channel (Health 1.0) among participants [20-22]. We selected eHL Scale (eHEALS) because it is the most widely used DHL scale in the world and is easy to answer for participants [23]. The J-eHEALS used a 5-point Likert scale to measure perceived eHL (from 1 [strongly disagree] to 5 [strongly agree]; score range=8-40). To validate the J-eHEALS, a confirmatory factor analysis was conducted using data from the survey [20]. We divided the J-eHEALS scores into 2 categories (high and low) relative to the median score.

Moreover, the Digital Health Literacy Instrument (DHLI) adapted to the COVID-19 pandemic was also used to evaluate eHL levels, including literacy for using social networking sites (SNSs)—such as Facebook (Meta Platforms, Inc) and Twitter (Twitter, Inc)—referred to as “Health 2.0” [15]. The DHLI was designed to assess eHL for Health 1.0 and Health 2.0 and is widely used throughout the world [14-18,22]. We used J-eHEALS and DHLI to evaluate eHL levels for both Health 1.0 and Health 2.0. The DHLI contains 7 subscales: information searching, adding self-generated content, evaluating reliability, determining relevance, operational skills, navigation skills, and protecting privacy. Each subscale included 3 items to be answered on a 4-point Likert scale (ranging from 1 [very difficult] to 4 [very easy]). The COVID-HL network used DHLI adapted to COVID-19 and did not use the subscales of operational and navigation DHLI skills adapted to COVID-19 [13]. However, we included these subscales because they were crucial to accessing health information and navigating the internet. Moreover, although a recent study developed the DHLI [24], data on these skills adapted to COVID-19 among adult internet users in Japan were lacking. We divided each subscale and the total score of the DHLI into 1 of 2 categories (high or low) relative to the median score based on previous studies [14-18]. We translated DHLI adapted to the COVID-19 to Japanese, back translated it, and then confirmed their authors (Multimedia Appendix 1).

#### Outcomes: Web-Based Health Information–Seeking Behavior

The measures of web-based health information–seeking behaviors on the COVID-19 pandemic were assessed using a list of 10 different web sources: search engines (such as Google [Google LLC], Bing [Microsoft Corp], and Yahoo! [Yahoo Inc]), websites of public authorities (such as Ministry of Health, Labour and Welfare and the Japan Medical Association), Wikipedia, web-based encyclopedias, SNSs (such as Facebook, Instagram, and Twitter), YouTube, blogs providing medicine- and health-related information, medicine- and health-related question and answer sites (such as Yahoo! Answers), medicine- and health-related information portals, websites run by physicians or medical facilities, and news portal sites (including information gathered from newspapers and TV stations). These items were answered using a 5-point scale (0=do not know, 1=never, 2=often, 3=rarely, 4=sometimes, and 5=often). They were then assigned to either a “do not know–rarely” or “sometimes–often” category.

Moreover, we asked the participants to indicate from a list of 10 topics what they were searching for regarding COVID-19: the prevalence (such as number of people infected), infection route, symptoms, preventive measures (including disinfection and handwashing), rules and behavior (such as disinfection), assessment of its current status (such as declarations, measures, and stages), recommendations (including information from the Ministry of Health, Labour and Welfare and municipal governments), refraining from specific actions (such as eating out, traveling, and commuting to work), the economic and social effects, dealing with the psychological stress it causes, and information concerning the vaccine (effectiveness, side effects, and vaccination status). Participants answered “yes” or “no” to these items.

#### Sociodemographic and Other Variables

Sociodemographic and other variables were included as covariates in this regression model used by prior studies that examined the factors associated with eHL (gender, age groups, equivalent income, education status, marital status, cigarette smoking, alcohol consumption, physical exercise habits, and conditions that could likely lead to severe COVID-19 illness) [20-22]. Equivalent income was estimated by dividing annual income by the square root of the number of families [25]. We divided the equivalent income into 12 categories (<1-≥10 million Yen and “not answered”). Education status was divided into 4 categories (≤high school graduate, 2-year college or career college, higher university education, and “not answered”). Regarding marital status, the participants who answered “married” were categorized as “married.” The participants who answered “never married,” “widowed,” or “divorced” were categorized as “not married.” Concerning health behaviors, we assessed 3 items related to smoking, alcohol consumption, and physical exercise. Regarding smoking status, responses such as “never” or “quit” were categorized as “no smoking” and “smoking” or “sometimes smoking” as “smoking.” Alcohol consumption was determined using “yes” or “no” responses and the quantity of alcohol consumed. The participants who answered “no” or “quit” were categorized as “no.” Participants who responded with an alcohol intake of <20 g at once were categorized as “<20 g/once,” and those who drank alcohol ≥20 g at once were categorized as “≥20 g/once.” The physical exercise of participants was assessed subjectively based on whether they performed a 30-minute physical exercise ≥2 times a week for a year or longer (“yes” or “no”). We selected 6 conditions (hypertension, diabetes, chronic obstructive pulmonary diseases, heart diseases, and chronic kidney diseases; BMI ≥30) to determine the possibility of becoming severely ill with COVID-19. “Yes” responses to ≥1 questions concerning the prevalence of conditions that were likely to cause severe illness with COVID-19 were categorized as “Yes.”

#### Difficulties in Seeking and Using COVID-19 Information

We asked the participants the descriptive open-ended question, “What difficulties did you have in seeking and using COVID-19-related information on the internet?” The item was in the required field and thus could not be left unanswered.

### Analysis Using a Mixed Methods Strategy

#### Overview

We used the concurrent triangulation design of mixed methods strategy to analyze both quantitative and qualitative data in the internet-based survey [26]. In mixed methods analyses, the use of complementary methods integrating quantitative and qualitative approaches to address a complex question can generate deeper insights than using either approach alone or both approaches separately [27]. Mixed methods research enables a more comprehensive understanding of the phenomenon under investigation by integrating both quantitative and qualitative data. Furthermore, findings can be validated across different data sets by using both quantitative and qualitative methods. The triangulation of data from multiple methods enhances the credibility and reliability of a study’s findings. By adopting a mixed methods approach, it is possible to attain a broader understanding of the association between eHL and web-based health information–seeking behaviors, as well as the difficulties encountered in seeking and using this information.

#### Qualitative Content Analysis of Qualitative Data

Descriptive responses regarding difficulties in seeking and using COVID-19 information were analyzed using the inductive qualitative content analysis approach [28-30]. The contents were inductively organized into codes and categories to achieve trustworthiness [29]. YT and SM performed the analysis. All the responses were read and interpreted repeatedly. After discussing the meanings of the responses, phrases or sentences were coded for the analysis. The coding frame was changed when new codes emerged, and sentences were reread using the new structure. This constant comparison process was also used to develop conceptualize the responses into broad categories after further discussion. We finally aggregated categories into themes. We used the MAXQDA Analytics Pro 2022 (version 22.4.1; VERBI Software GmbH) for qualitative content analysis.

#### Statistical Analysis of Quantitative Data

First, a chi-square test was performed to compare the proportion of participants with low and high eHL by assessing the eHEALS and subscales of the DHLI. The internal consistencies of the subscales and the total scale were assessed using Cronbach α. We then examined the association of eHL levels with using web sources of COVID-19 information by using a multivariable logistic regression model that adjusted for all covariates. In addition, the associations of eHL levels with searching for specific COVID-19 topics were examined using a multivariable logistic regression model that adjusted for all covariates. Adjusted odds ratios (AORs) and 95% CIs were estimated. We explored the relationship between eHL and categories of difficulties more thoroughly. eHEALS and DHLI total scores were classified into quartiles to observe variations in dose response, followed by the performance of the Cochrane-Armitage test for trend analysis. Two-tailed *P* values <.05 were considered significant. All analyses were conducted using SPSS (version 28.0; IBM Corp).

## Results

### Study Participant Selection

**Figure 1:**
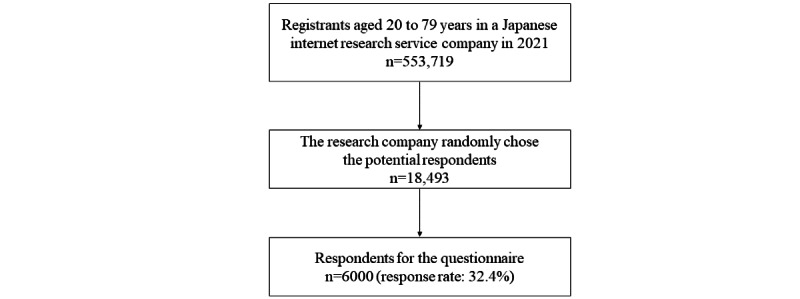
Flowchart of patient selection in this study.

Figure 1 illustrates this study’s participant selection process. The research company chose 18,493 potential respondents in October 2021, and 6000 responses were obtained from respondents who provided complete information for the study variables (response rate: 32.4%).

### Characteristics of Study Participants

The proportions of each gender and age group were identical (Table 1). The proportion of participants whose equivalent income was 3 to 4 million Yen was 19.98% (1199/6000). About 48.45% (2907/6000) of the participants had graduated from university or had higher education, and 54.67% (3280/6000) were married. Approximately 16.13% (968/6000) of the participants reported a cigarette smoking habit, 61.45% (3687/6000) consumed alcohol, and 32.78% (1967/6000) exercised regularly. Moreover, 23.02% (1381/6000) of the respondents had ≥1 health conditions likely to lead to severe COVID-19 illness.

**Table 1:** Characteristics of participants.

Characteristics		Participants (N=6000), n (%)
**Gender**		
	Men	3000 (50)
	Women	3000 (50)
**Age groups (y)**		
	20-29	1000 (16.67)
	30-39	1000 (16.67)
	40-49	1000 (16.67)
	50-59	1000 (16.67)
	60-69	1000 (16.67)
	≥70	1000 (16.67)
**Equivalent income (million Y** **en** **; Y** **en 1=US $ 0.0088)**		
	<1	413 (6.88)
	1-<2	878 (14.63)
	2-<3	972 (16.2)
	3-<4	1199 (19.98)
	4-<5	852 (14.2)
	5-<6	486 (8.1)
	6-<7	430 (7.17)
	7-<8	165 (2.75)
	8-<9	70 (1.17)
	9-<10	72 (1.2)
	≥10	107 (1.78)
	Not answered	356 (5.93)
**Education status**		
	≤High school	1768 (29.47)
	2-year college or career college	1298 (21.63)
	University or higher education	2907 (48.45)
	No answer	27 (0.45)
**Marital status**		
	No	2683 (44.72)
	Yes	3280 (54.67)
	Not answered	37 (0.62)
**Cigarette smoking**		
	No	5032 (83.87)
	Yes	968 (16.13)
**Alcohol consumption**		
	No	2313 (38.55)
	<20 g/once	2053 (34.22)
	≥20 g/once	1634 (27.23)
**Physical exercise habit**		
	No	4033 (67.22)
	Yes	1967 (32.78)
**Conditions that could likely lead to severe COVID-19 illness**		
	No	4619 (76.98)
	Yes	1381 (23.02)

### Scores and Internal Consistencies of the DHLI Among This Study’s Participants

Table 2 presents the DHLI scores and internal consistencies among the study participants. The 7 subscales’ internal consistencies (Cronbach α) ranged from acceptable to good (0.83-0.94). Moreover, the mean DHLI total score was 3.08 (SD 0.49), and Cronbach α of the complete scale was 0.92.

**Table 2:** Scores and internal consistencies of the DHLI^a,b^.

Subscales and total score of the DHLI	Values, mean (SD)	Values, median (IQR)	Cronbach α
Information search	3.01 (0.61)	3.0 (2.7-3.3)	0.91
Adding self-generated information	2.73 (0.73)	3.0 (2.0-3.0)	0.94
Evaluating reliability	2.66 (0.65)	2.7 (2.0-3.0)	0.88
Determining relevance	2.87 (0.59)	3.0 (2.7-3.0)	0.90
Operational skills	3.31 (0.62)	3.0 (3.0-4.0)	0.88
Navigation skills	3.59 (0.73)	4.0 (3.3-4.0)	0.83
Privacy protection	3.42 (0.91)	4.0 (3.0-4.0)	0.87
Total score	3.08 (0.49)	3.1 (2.8-3.4)	0.92

^a^DHLI: Digital Health Literacy Instrument.

^b^The subscale scores and total DHLI score range from 0 to 4.

### Differences of Characteristics by eHL From eHEALS and DHLI Subscales

Compared to those with low eHEALS, participants with high eHEALS were more likely to be older (*P*<.001), have higher income (*P*<.001) and education levels (*P*<.001), and be married (*P*=.007; Tables 3 and 4). Moreover, they were more likely to consume alcohol (*P*=.02) and have physical exercise habits (*P*<.001) and conditions leading to severe COVID-19 illness (*P*=.004) than those with low eHEALS.

Among the participants with high total DHLI scores, there were higher proportions of men (*P*=.002), those aged 20 to 39 years (*P*<.001), those with higher equivalent income (*P*<.001), and those with higher education status (*P*<.001). They were more likely to consume alcohol (*P*=.02) and have physical exercise habits (*P*<.001) and less likely to have conditions that could lead to severe COVID-19 illness (*P*<.001). In addition, participants with higher subscores of DHLI generally consisted of higher proportions of men, had higher equivalent income and higher education status, and were more likely to be married. They were more likely to consume alcohol and less likely to have conditions that could lead to severe COVID-19 illness. Moreover, participants with higher scores on information searching, adding self-generated content, evaluating reliability, determining relevance, and operational skills were more likely to have an exercise habit. However, participants with higher scores on navigation skills and privacy protection were less likely to have an exercise habit.

**Table 3:** Differences of characteristics based on the scores of eHEALS^a^, DHLI^b^, and its subscales.

Characteristic			eHEALS								Information searching									Adding self-generated information									Evaluation reliability					
			Low (n=2228), n (%)		High (n=3772), n (%)		*P* value^c^			Low (n=1613), n (%)			High (n=4387), n (%)		*P* value^c^			Low (n=2702), n (%)				High (n=3298), n (%)		*P* value^c^			Low (n=2475), n (%)				High (n=3525), n (%)		*P* value^c^	
**Gender**								.24								.47									.04									<.001
	Men	1136 (50.99)		1864 (49.42)					794 (49.23)			2206 (50.28)					1311 (48.52)				1689 (51.21)					1135 (45.86)				1865 (52.91)				
	Women	1092 (49.01)		1908 (50.58)					819 (50.77)			2181 (49.72)					1391 (51.48)				1609 (48.79)					1340 (54.14)				1660 (47.09)				
**Age groups (y)**								<.001								.83									.17									<.001
	20-29	421 (18.9)		579 (15.35)					272 (16.86)			728 (16.59)					420 (15.54)				580 (17.59)					362 (14.63)				638 (18.1)				
	30-39	376 (16.88)		624 (16.54)					268 (16.62)			732 (16.69)					453 (16.77)				547 (16.59)					389 (15.72)				611 (17.33)				
	40-49	374 (16.79)		626 (16.6)					271 (16.8)			729 (16.62)					480 (17.76)				520 (15.77)					405 (16.36)				595 (16.88)				
	50-59	387 (17.37)		613 (16.25)					256 (15.87)			744 (16.96)					442 (16.36)				558 (16.92)					424 (17.13)				576 (16.34)				
	60-69	355 (15.93)		645 (17.1)					263 (16.31)			737 (16.8)					456 (16.88)				544 (16.49)					422 (17.05)				578 (16.4)				
	≥70	315 (14.14)		685 (18.16)					283 (17.54)			717 (16.34)					451 (16.69)				549 (16.65)					473 (19.11)				527 (14.95)				
**Equivalent income (million Yen; Y** **en 1=US $ 0.0088)**								<.001								<.001									<.001									<.001
	<1	177 (7.94)		236 (6.26)					131 (8.12)			282 (6.43)					202 (7.48)				211 (6.4)					182 (7.35)				231 (6.55)				
	1-<2	375 (16.83)		503 (13.34)					279 (17.3)			599 (13.65)					468 (17.32)				410 (12.43)					433 (17.49)				445 (12.62)				
	2-<3	394 (17.68)		578 (15.32)					270 (16.74)			702 (16)					468 (17.32)				504 (15.28)					439 (17.74)				533 (15.12)				
	3-<4	437 (19.61)		762 (20.2)					320 (19.84)			879 (20.04)					537 (19.87)				662 (20.07)					491 (19.84)				708 (20.09)				
	4-<5	300 (13.46)		552 (14.63)					208 (12.9)			644 (14.68)					366 (13.55)				486 (14.74)					336 (13.58)				516 (14.64)				
	5-<6	166 (7.45)		320 (8.48)					110 (6.82)			376 (8.57)					189 (6.99)				297 (9.01)					177 (7.15)				309 (8.77)				
	6-<7	130 (5.83)		300 (7.95)					93 (5.77)			337 (7.68)					141 (5.22)				289 (8.76)					145 (5.86)				285 (8.09)				
	7-<8	45 (2.02)		120 (3.18)					33 (2.05)			132 (3.01)					58 (2.15)				107 (3.24)					48 (1.94)				117 (3.32)				
	8-<9	24 (1.08)		46 (1.22)					15 (0.93)			55 (1.25)					28 (1.04)				42 (1.27)					18 (0.73)				52 (1.48)				
	9-<10	24 (1.08)		48 (1.27)					14 (0.87)			58 (1.32)					16 (0.59)				56 (1.7)					14 (0.57)				58 (1.65)				
	≥10	17 (0.76)		90 (2.39)					12 (0.74)			95 (2.17)					27 (1)				80 (2.43)					18 (0.73)				89 (2.52)				
	Not answered	139 (6.24)		217 (5.75)					128 (7.94)			228 (5.2)					202 (7.48)				154 (4.67)					174 (7.03)				182 (5.16)				
**Education status**								<.001								.001									<.001									<.001
	≤High school	725 (32.54)		1043 (27.65)					528 (32.73)			1240 (28.27)					879 (32.53)				889 (26.96)					852 (34.42)				916 (25.99)				
	2-year college or career college	443 (19.88)		855 (22.67)					321 (19.9)			977 (22.27)					590 (21.84)				708 (21.47)					521 (21.05)				777 (22.04)				
	≥University	1053 (47.26)		1854 (49.15)					752 (46.62)			2155 (49.12)					1216 (45)				1691 (51.27)					1087 (43.92)				1820 (51.63)				
	Not answered	7 (0.31)		20 (0.53)					12 (0.74)			15 (0.34)					17 (0.63)				10 (0.3)					15 (0.61)				12 (0.34)				
**Marital status**								.007								.002									.03									.14
	No	1049 (47.08)		1634 (43.32)					774 (47.99)			1909 (43.51)					1253 (46.37)				1430 (43.36)					1094 (44.2)				1589 (45.08)				
	Yes	1170 (52.51)		2110 (55.94)					825 (51.15)			2455 (55.96)					1429 (52.89)				1851 (56.12)					1360 (54.95)				1920 (54.47)				
	Not answered	9 (0.4)		28 (0.74)					14 (0.87)			23 (0.52)					20 (0.74)				17 (0.52)					21 (0.85)				16 (0.45)				
**Cigarette smoking**								.63								.13									<.001									<.001
	No	1862 (83.57)		3170 (84.04)					1372 (85.06)			3660 (83.43)					2329 (86.2)				2703 (81.96)					2129 (86.02)				2903 (82.35)				
	Yes	366 (16.43)		602 (15.96)					241 (14.94)			727 (16.57)					373 (13.8)				595 (18.04)					346 (13.98)				622 (17.65)				
**Alcohol consumption**								.02								<.001									<.001									<.001
	No	911 (40.89)		1402 (37.17)					680 (42.16)			1633 (37.22)					1117 (41.34)				1196 (36.26)					1031 (41.66)				1282 (36.37)				
	<20 g/once	731 (32.81)		1322 (35.05)					555 (34.41)			1498 (34.15)					935 (34.6)				1118 (33.9)					856 (34.59)				1197 (33.96)				
	≥20 g/once	586 (26.3)		1048 (27.78)					378 (23.43)			1256 (28.63)					650 (24.06)				984 (29.84)					588 (23.76)				1046 (29.67)				
**Physical exercise habit**								<.001								<.001									<.001									<.001
	No	1683 (75.54)		2350 (62.3)					1157 (71.73)			2876 (65.56)					1957 (72.43)				2076 (62.95)					1781 (71.96)				2252 (63.89)				
	Yes	545 (24.46)		1422 (37.7)					456 (28.27)			1511 (34.44)					745 (27.57)				1222 (37.05)					694 (28.04)				1273 (36.11)				
**Conditions leading to severe COVID-19 illness**								.004								.004									.24									.16
	No	1745 (78.32)		2874 (76.19)					1200 (74.4)			3419 (77.93)					2061 (76.28)				2558 (77.56)					1883 (76.08)				2736 (77.62)				
	Yes	483 (21.68)		898 (23.81)					413 (25.6)			968 (22.07)					641 (23.72)				740 (22.44)					592 (23.92)				789 (22.38)				

^a^eHEALS: eHealth Literacy Scale.

^b^DHLI: Digital Health Literacy Instrument.

^c^The chi-square test.

**Table 4:** Differences of characteristics based on the scores of eHEALS^a^, DHLI^b^, and its subscales (continued).

Characteristic		Determining relevance					Operational skills						Navigation skills							Privacy protection						Total score			
		Low (n=2051), n (%)	High (n=3949), n (%)	*P* value^c^		Low (n=1076), n (%)		High (n=4924), n (%)	*P* value^c^		Low (n=1905), n (%)			High (n=4095), n (%)	*P* value^c^			Low (n=2206), n (%)			High (n=3794), n (%)	*P* value^c^		Low (n=3264), n (%)			High (n=2736), n (%)	*P* value^c^	
**Gender**					.17					<.001							.002						.75						.002
	Men	1051 (51.24)	1949 (49.35)			455 (42.29)		2545 (51.69)			896 (47.03)			2104 (51.38)				1109 (50.27)			1891 (49.84)			1572 (48.16)			1428 (52.19)		
	Women	1000 (48.76)	2000 (50.65)			621 (57.71)		2379 (48.31)			1009 (52.97)			1991 (48.62)				1097 (49.73)			1903 (50.16)			1692 (51.84)			1308 (47.81)		
**Age groups (y)**					.73					<.001							<.001						<.001						<.001
	20-29	339 (16.53)	661 (16.74)			159 (14.78)		841 (17.08)			313 (16.43)			687 (16.78)				378 (17.14)			622 (16.39)			500 (15.32)			500 (18.27)		
	30-39	334 (16.28)	666 (16.87)			163 (15.15)		837 (17)			307 (16.12)			693 (16.92)				349 (15.82)			651 (17.16)			514 (15.75)			486 (17.76)		
	40-49	359 (17.5)	641 (16.23)			147 (13.66)		853 (17.32)			269 (14.12)			731 (17.85)				323 (14.64)			677 (17.84)			527 (16.15)			473 (17.29)		
	50-59	330 (16.09)	670 (16.97)			168 (15.61)		832 (16.9)			298 (15.64)			702 (17.14)				340 (15.41)			660 (17.4)			535 (16.39)			465 (17)		
	60-69	337 (16.43)	663 (16.79)			204 (18.96)		796 (16.17)			303 (15.91)			697 (17.02)				356 (16.14)			644 (16.97)			559 (17.13)			441 (16.12)		
	≥70	352 (17.16)	648 (16.41)			235 (21.84)		765 (15.54)			415 (21.78)			585 (14.29)				460 (20.85)			540 (14.23)			629 (19.27)			371 (13.56)		
**Equivalent income (million Yen; Yen 1=US $0.0088)**					<.001					<.001							.005						.01						<.001
	<1	159 (7.75)	254 (6.43)			98 (9.11)		315 (6.4)			149 (7.82)			264 (6.45)				155 (7.03)			258 (6.8)			246 (7.54)			167 (6.1)		
	1-<2	340 (16.58)	538 (13.62)			208 (19.33)		670 (13.61)			296 (15.54)			582 (14.21)				339 (15.37)			539 (14.21)			542 (16.61)			336 (12.28)		
	2-<3	340 (16.58)	632 (16)			187 (17.38)		785 (15.94)			312 (16.38)			660 (16.12)				382 (17.32)			590 (15.55)			559 (17.13)			413 (15.1)		
	3-<4	418 (20.38)	781 (19.78)			201 (18.68)		998 (20.27)			393 (20.63)			806 (19.68)				470 (21.31)			729 (19.21)			660 (20.22)			539 (19.7)		
	4-<5	293 (14.29)	559 (14.16)			136 (12.64)		716 (14.54)			280 (14.7)			572 (13.97)				316 (14.32)			536 (14.13)			458 (14.03)			394 (14.4)		
	5-<6	137 (6.68)	349 (8.84)			70 (6.51)		416 (8.45)			141 (7.4)			345 (8.42)				154 (6.98)			332 (8.75)			224 (6.86)			262 (9.58)		
	6-<7	127 (6.19)	303 (7.67)			47 (4.37)		383 (7.78)			117 (6.14)			313 (7.64)				141 (6.39)			289 (7.62)			198 (6.07)			232 (8.48)		
	7-<8	42 (2.05)	123 (3.11)			16 (1.49)		149 (3.03)			39 (2.05)			126 (3.08)				50 (2.27)			115 (3.03)			65 (1.99)			100 (3.65)		
	8-<9	18 (0.88)	52 (1.32)			8 (0.74)		62 (1.26)			22 (1.15)			48 (1.17)				26 (1.18)			44 (1.16)			35 (1.07)			35 (1.28)		
	9-<10	8 (0.39)	64 (1.62)			4 (0.37)		68 (1.38)			15 (0.79)			57 (1.39)				21 (0.95)			51 (1.34)			25 (0.77)			47 (1.72)		
	≥10	20 (0.98)	87 (2.2)			9 (0.84)		98 (1.99)			23 (1.21)			84 (2.05)				38 (1.72)			69 (1.82)			30 (0.92)			77 (2.81)		
	Not answered	149 (7.26)	207 (5.24)			92 (8.55)		264 (5.36)			118 (6.19)			238 (5.81)				114 (5.17)			242 (6.38)			222 (6.8)			134 (4.9)		
**Education status**					.007					<.001							.002						.06						<.001
	≤High school	655 (31.94)	1113 (28.18)			451 (41.91)		1317 (26.75)			619 (32.49)			1149 (28.06)				618 (28.01)			1150 (30.31)			1053 (32.26)			715 (26.13)		
	2-year college or career college	422 (20.58)	876 (22.18)			248 (23.05)		1050 (21.32)			410 (21.52)			888 (21.68)				460 (20.85)			838 (22.09)			707 (21.66)			591 (21.6)		
	≥University	961 (46.86)	1946 (49.28)			364 (33.83)		2543 (51.65)			871 (45.72)			2036 (49.72)				1116 (50.59)			1791 (47.21)			1487 (45.56)			1420 (51.9)		
	Not answered	13 (0.63)	14 (0.35)			13 (1.21)		14 (0.28)			5 (0.26)			22 (0.54)				12 (0.54)			15 (0.4)			17 (0.52)			10 (0.37)		
**Marital status**					.04					<.001							.99						.58						.44
	No	913 (44.51)	1770 (44.82)			454 (42.19)		2229 (45.27)			850 (44.62)			1833 (44.76)				967 (43.83)			1716 (45.23)			1460 (44.73)			1223 (44.7)		
	Yes	1118 (54.51)	2162 (54.75)			600 (55.76)		2680 (54.43)			1043 (54.75)			2237 (54.63)				1225 (55.53)			2055 (54.16)			1780 (54.53)			1500 (54.82)		
	Not answered	20 (0.98)	17 (0.43)			22 (2.04)		15 (0.3)			12 (0.63)			25 (0.61)				14 (0.63)			23 (0.61)			24 (0.74)			13 (0.48)		
**Cigarette smoking**					.61					.18							.06						.56						.15
	No	1727 (84.2)	3305 (83.69)			917 (85.22)		4115 (83.57)			1573 (82.57)			3459 (84.47)				1842 (83.5)			3190 (84.08)			2758 (84.5)			2274 (83.11)		
	Yes	324 (15.8)	644 (16.31)			159 (14.78)		809 (16.43)			332 (17.43)			636 (15.53)				364 (16.5)			604 (15.92)			506 (15.5)			462 (16.89)		
**Alcohol consumption**					.008					<.001							.05						.02						.008
	No	844 (41.15)	1469 (37.2)			507 (47.12)		1806 (36.68)			721 (37.85)			1592 (38.88)				801 (36.31)			1512 (39.85)			1303 (39.92)			1010 (36.92)		
	<20 g/once	685 (33.4)	1368 (34.64)			321 (29.83)		1732 (35.17)			627 (32.91)			1426 (34.82)				785 (35.58)			1268 (33.42)			1122 (34.38)			931 (34.03)		
	≥20 g/once	522 (25.45)	1112 (28.16)			248 (23.05)		1386 (28.15)			557 (29.24)			1077 (26.3)				620 (28.11)			1014 (26.73)			839 (25.7)			795 (29.06)		
**Physical exercise habit**					<.001					.02							.01						<.001						<.001
	No	1494 (72.84)	2539 (64.29)			755 (70.17)		3278 (66.57)			1238 (64.99)			2795 (68.25)				1413 (64.05)			2620 (69.06)			2276 (69.73)			1757 (64.22)		
	Yes	557 (27.16)	1410 (35.71)			321 (29.83)		1646 (33.43)			667 (35.01)			1300 (31.75)				793 (35.95)			1174 (30.94)			988 (30.27)			979 (35.78)		
**Conditions that could lead to severe COVID-19 illness**					.02					.01							<.001						<.001						<.001
	No	1544 (75.28)	3075 (77.87)			797 (74.07)		3822 (77.62)			1397 (73.33)			3222 (78.68)				1603 (72.67)			3016 (79.49)			2424 (74.26)			2195 (80.23)		
	Yes	507 (24.72)	874 (22.13)			279 (25.93)		1102 (22.38)			508 (26.67)			873 (21.32)				603 (27.33)			778 (20.51)			840 (25.74)			541 (19.77)		

^a^eHEALS: eHealth Literacy Scale.

^b^DHLI: Digital Health Literacy Instrument.

^c^The chi-square test.

### Associations of eHL With Using Web Sources for Finding COVID-19 Information

Table 5 illustrates the proportion of “sometimes” or “often” responses to questions on using each web source. The most common web sources were search engines (4614/6000, 76.9%), followed by news portal sites (3350/6000, 55.83%). Participants with high eHEALS were more likely to use all web sources of information about COVID-19 than those with low eHEALS (Tables 6 and 7). The participants with high scores on the DHLI subscales information searching, adding self-generated information, evaluating reliability, determining relevance, and operational skills were also more likely to search for COVID-19 information using all web sources than participants with low scores on these subscales. Participants with high navigation skill scores were more likely to use search engines but less likely to use YouTube to search for COVID-19 information (AOR 0.88, 95% CI 0.79-0.99). Moreover, participants with high privacy protection scores were less likely to use websites of public authorities (AOR 0.80, 95% CI 0.72-0.89), Wikipedia (AOR 0.82, 95% CI 0.74-0.92), SNSs (AOR 0.74, 95% CI 0.66-0.83), YouTube (AOR 0.84, 95% CI 0.75-0.94), blogs providing medicine- and health-related information (AOR 0.81, 95% CI 0.72-0.92), question and answer sites (AOR 0.75, 95% CI 0.67-0.85), medicine- and health-related information portals (AOR 0.75, 95% CI 0.67-0.85), and websites run by physicians or medical facilities (AOR 0.72, 95% CI 0.64-0.81) for finding COVID-19 information. In addition, participants with high total DHLI scores were more likely to use all web sources of COVID-19 information than those with low total scores.

**Table 5:** The proportion of “sometimes” or “often” responses to questions on using each web source.

Web source	Participants (N=6000), n (%)
Search engines	4614 (76.9)
News portal sites	3350 (55.83)
Websites of public authorities	2652 (44.2)
Wikipedia, web-based encyclopedias	2232 (37.2)
Social media sites	2088 (34.8)
YouTube	2080 (34.67)
Websites run by physicians or medical facilities	1718 (28.63)
Question and answer sites related to medicine and health	1717 (28.62)
Medicine and health-related information portals	1623 (27.05)
Blogs providing medicine and health-related information	1379 (22.98)

**Table 6:** Associations of eHL^a^ levels with using web sources for finding COVID-19 information.

eHL		Search engines			Websites of public authorities			Wikipedia and web-based encyclopedias			SNSs^b^			YouTube	
		Value, n (%)	AOR^c^ (95% CI)^d^	Value, n (%)		AOR (95% CI)^d^	Value, n (%)		AOR (95% CI)^d^	Value, n (%)		AOR (95% CI)^d^	Value, n (%)		AOR (95% CI)^d^
**eHEALS^e^**															
	Low (n=2228)	1583 (71.05)	1.00 (reference)	748 (33.57)		1.00 (reference)	641 (28.77)		1.00 (reference)	640 (28.73)		1.00 (reference)	638 (28.64)		1.00 (reference)
	High (n=3772)	3031 (80.36)	1.57 (1.38-1.78)	1904 (50.48)		1.88 (1.68-2.11)	1591 (42.18)		1.70 (1.51-1.90)	1448 (38.39)		1.61 (1.43-1.81)	1442 (38.23)		1.50 (1.34-1.69)
**Information search**															
	Low (n=1613)	1133 (70.24)	1.00 (reference)	591 (36.64)		1.00 (reference)	454 (28.15)		1.00 (reference)	466 (28.89)		1.00 (reference)	452 (28.02)		1.00 (reference)
	High (n=4387)	3481 (79.35)	1.54 (1.35-1.76)	2061 (46.98)		1.44 (1.27-1.62)	1778 (40.53)		1.66 (1.46-1.89)	1622 (36.97)		1.41 (1.24-1.60)	1628 (37.11)		1.47 (1.30-1.67)
**Adding self-generated information**															
	Low (n=2702)	1992 (73.72)	1.00 (reference)	1034 (38.27)		1.00 (reference)	813 (30.09)		1.00 (reference)	786 (29.09)		1.00 (reference)	771 (28.53)		1.00 (reference)
	High (n=3298)	2622 (79.5)	1.29 (1.14-1.46)	1618 (49.06)		1.42 (1.27-1.58)	1419 (43.03)		1.64 (1.47-1.84)	1302 (39.48)		1.51 (1.35-1.70)	1309 (39.69)		1.58 (1.41-1.76)
**Evaluating reliability**															
	Low (n=2475)	1849 (74.71)	1.00 (reference)	934 (37.74)		1.00 (reference)	747 (30.18)		1.00 (reference)	722 (29.17)		1.00 (reference)	718 (29.01)		1.00 (reference)
	High (n=3525)	2765 (78.44)	1.16 (1.03-1.32)	1718 (48.74)		1.45 (1.30-1.61)	1485 (42.13)		1.56 (1.39-1.74)	1366 (38.75)		1.39 (1.24-1.56)	1362 (38.64)		1.44 (1.29-1.62)
**Determining relevance**															
	Low (n=2051)	1447 (70.55)	1.00 (reference)	760 (37.06)		1.00 (reference)	617 (30.08)		1.00 (reference)	603 (29.4)		1.00 (reference)	599 (29.21)		1.00 (reference)
	High (n=3949)	3167 (80.2)	1.60 (1.41-1.82)	1892 (47.91)		1.46 (1.30-1.63)	1615 (40.9)		1.53 (1.37-1.72)	1485 (37.6)		1.39 (1.23-1.57)	1481 (37.5)		1.40 (1.25-1.58)
**Operational skills**															
	Low (n=1076)	658 (61.15)	1.00 (reference)	310 (28.81)		1.00 (reference)	263 (24.44)		1.00 (reference)	260 (24.16)		1.00 (reference)	322 (29.93)		1.00 (reference)
	High (n=4924)	3956 (80.34)	2.43 (2.09-2.82)	2342 (47.56)		2.07 (1.78-2.40)	1969 (39.99)		1.90 (1.62-2.21)	1828 (37.12)		1.67 (1.42-1.96)	1758 (35.7)		1.22 (1.05-1.41)
**Navigation skills**															
	Low (n=1905)	1436 (75.38)	1.00 (reference)	845 (44.36)		1.00 (reference)	692 (36.33)		1.00 (reference)	643 (33.75)		1.00 (reference)	698 (36.64)		1.00 (reference)
	High (n=4095)	3178 (77.61)	1.15 (1.01-1.31)	1807 (44.13)		1.00 (0.89-1.12)	1540 (37.61)		1.05 (0.94-1.18)	1445 (35.29)		1.06 (0.94-1.20)	1382 (33.75)		0.88 (0.79-0.99)
**Protecting privacy**															
	Low (n=2206)	1706 (77.33)	1.00 (reference)	1061 (48.1)		1.00 (reference)	891 (40.39)		1.00 (reference)	856 (38.8)		1.00 (reference)	826 (37.44)		1.00 (reference)
	High (n=3794)	2908 (76.65)	1.01 (0.88-1.14)	1591 (41.93)		0.80 (0.72-0.89)	1341 (35.35)		0.82 (0.74-0.92)	1232 (32.47)		0.74 (0.66-0.83)	1254 (33.05)		0.84 (0.75-0.94)
**Total score of DHLI^f^**															
	Low (n=3264)	2426 (74.33)	1.00 (reference)	1302 (39.89)		1.00 (reference)	1065 (32.63)		1.00 (reference)	997 (30.55)		1.00 (reference)	1040 (31.86)		1.00 (reference)
	High (n=2736)	2188 (79.97)	1.34 (1.18-1.52)	1350 (49.34)		1.38 (1.24-1.54)	1167 (42.65)		1.46 (1.31-1.63)	1091 (39.88)		1.39 (1.24-1.56)	1040 (38.01)		1.25 (1.12-1.40)

^a^eHL: eHealth literacy.

^b^SNS: social networking site.

^c^AOR: adjusted odds ratio.

^d^Multivariable logistic regression analysis adjusted for all covariates (ie, gender, age groups, equivalent income, education status, marital status, cigarette smoking, alcohol consumption, physical exercise habit, and conditions leading to severe illness due to COVID-19).

^e^eHEALS: eHealth Literacy Scale.

^f^DHLI: Digital Health Literacy Instrument.

**Table 7:** Associations of eHL^a^ levels with using web sources for finding COVID-19 information (continued).

eHL		Blogs providing medicine- and health-related information		Medicine- and health-related question and answer sites		Medicine- and health-related information portals		Websites run by physicians or medical facilities		News portal sites	
		Value, n (%)	AOR^b^ (95% CI)^c^	Value, n (%)	AOR (95% CI)^c^	Value, n (%)	AOR (95% CI)^c^	Value, n (%)	AOR (95% CI)^c^	Value, n (%)	AOR (95% CI)^c^
**eHEALS^d^**											
	Low (n=2228)	292 (13.1)	1.00 (reference)	417 (18.72)	1.00 (reference)	336 (15.08)	1.00 (reference)	375 (16.83)	1.00 (reference)	1045 (46.9)	1.00 (reference)
	High (n=3772)	1087 (28.82)	2.46 (2.13-2.84)	1300 (34.46)	2.08 (1.83-2.36)	1287 (34.12)	2.65 (2.31-3.04)	1343 (35.6)	2.51 (2.20-2.87)	2305 (61.11)	1.65 (1.48-1.85)
**Information search**											
	Low (n=1613)	232 (14.38)	1.00 (reference)	332 (20.58)	1.00 (reference)	305 (18.91)	1.00 (reference)	336 (20.83)	1.00 (reference)	789 (48.92)	1.00 (reference)
	High (n=4387)	1147 (26.15)	2.00 (1.71-2.35)	1385 (31.57)	1.71 (1.49-1.97)	1318 (30.04)	1.73 (1.50-2.00)	1382 (31.5)	1.65 (1.44-1.90)	2561 (58.38)	1.40 (1.24-1.57)
**Adding self-generated information**											
	Low (n=2702)	444 (16.43)	1.00 (reference)	610 (22.58)	1.00 (reference)	546 (20.21)	1.00 (reference)	591 (21.87)	1.00 (reference)	1419 (52.52)	1.00 (reference)
	High (n=3298)	935 (28.35)	1.87 (1.64-2.13)	1107 (33.57)	1.64 (1.45-1.85)	1077 (32.66)	1.75 (1.55-1.98)	1127 (34.17)	1.71 (1.52-1.93)	1931 (58.55)	1.19 (1.07-1.33)
**Evaluating reliability**											
	Low (n=2475)	378 (15.27)	1.00 (reference)	550 (22.22)	1.00 (reference)	481 (19.43)	1.00 (reference)	527 (21.29)	1.00 (reference)	1268 (51.23)	1.00 (reference)
	High (n=3525)	1001 (28.4)	2.07 (1.81-2.37)	1167 (33.11)	1.70 (1.50-1.92)	1142 (32.4)	1.85 (1.63-2.10)	1191 (33.79)	1.75 (1.55-1.98)	2082 (59.06)	1.33 (1.20-1.49)
**Determining relevance**											
	Low (n=2051)	336 (16.38)	1.00 (reference)	466 (22.72)	1.00 (reference)	407 (19.84)	1.00 (reference)	447 (21.79)	1.00 (reference)	1023 (49.88)	1.00 (reference)
	High (n=3949)	1043 (26.41)	1.71 (1.49-1.97)	1251 (31.68)	1.48 (1.30-1.68)	1216 (30.79)	1.66 (1.46-1.90)	1271 (32.19)	1.58 (1.39-1.80)	2327 (58.93)	1.37 (1.23-1.53)
**Operational skills**											
	Low (n=1076)	162 (15.06)	1.00 (reference)	222 (20.63)	1.00 (reference)	186 (17.29)	1.00 (reference)	204 (18.96)	1.00 (reference)	439 (40.8)	1.00 (reference)
	High (n=4924)	1217 (24.72)	1.78 (1.48-2.14)	1495 (30.36)	1.67 (1.41-1.97)	1437 (29.18)	1.86 (1.56-2.21)	1514 (30.75)	1.76 (1.49-2.09)	2911 (59.12)	2.01 (1.75-2.32)
**Navigation skills**											
	Low (n=1905)	430 (22.57)	1.00 (reference)	569 (29.87)	1.00 (reference)	530 (27.82)	1.00 (reference)	527 (27.66)	1.00 (reference)	1049 (55.07)	1.00 (reference)
	High (n=4095)	949 (23.17)	1.06 (0.93-1.21)	1148 (28.03)	0.95 (0.84-1.07)	1093 (26.69)	0.96 (0.85-1.09)	1191 (29.08)	1.07 (0.95-1.21)	2301 (56.19)	1.06 (0.94-1.19)
**Protecting privacy**											
	Low (n=2206)	569 (25.79)	1.00 (reference)	727 (32.96)	1.00 (reference)	687 (31.14)	1.00 (reference)	732 (33.18)	1.00 (reference)	1281 (58.07)	1.00 (reference)
	High (n=3794)	810 (21.35)	0.81 (0.72-0.92)	990 (26.09)	0.75 (0.67-0.85)	936 (24.67)	0.75 (0.67-0.85)	986 (25.99)	0.72 (0.64-0.81)	2069 (54.53)	0.90 (0.81-1.01)
**Total DHLI^e^ score**											
	Low (n=3264)	606 (18.57)	1.00 (reference)	830 (25.43)	1.00 (reference)	738 (22.61)	1.00 (reference)	799 (24.48)	1.00 (reference)	1691 (51.81)	1.00 (reference)
	High (n=2736)	773 (28.25)	1.66 (1.47-1.89)	887 (32.42)	1.39 (1.24-1.57)	885 (32.35)	1.56 (1.39-1.76)	919 (33.59)	1.47 (1.31-1.65)	1659 (60.64)	1.43 (1.29-1.60)

^a^eHL: eHealth literacy.

^b^AOR: adjusted odds ratio.

^c^Multivariable logistic regression analysis adjusted for all covariates (ie, gender, age groups, equivalent income, education status, marital status, cigarette smoking, alcohol consumption, physical exercise habit, and conditions leading to severe illness due to COVID-19).

^d^eHEALS: eHealth Literacy Scale.

^e^DHLI: Digital Health Literacy Instrument.

### Associations of eHL Levels With Searching Specific COVID-19 Topics

The most commonly searched specific COVID-19 topics were infectivity (4015/6000, 66.92%), followed by information about vaccine (3650/6000, 60.83%; Table 8). Participants with high eHEALS were more likely to search for all COVID-19-related topics than participants with low eHEALS (Tables 9 and 10). Moreover, participants with high total DHLI scores were more likely to search for information concerning infectivity and economic and social effects. In addition, participants with higher subscores of DHLI generally were more likely to search for information on the route of infection, assessment, economic and social effects, dealing with psychological stress, and the vaccine. However, the odds of searching for information on the route of infection and refraining from specific behaviors among participants with high navigation skills scores were 0.77 times (95% CI 0.67-0.89) and 0.88 times (95% CI 0.78-0.99) lower, respectively, than those among participants with lower scores. In addition, participants with high privacy protection scores were less likely to search for information on the route of infection (AOR 0.71, 95% CI 0.63-0.82), symptoms (AOR 0.81, 95% CI 0.73-0.90), preventive measures (AOR 0.74, 95% CI 0.66-0.83), rules and behaviors (AOR 0.87, 95% CI 0.77-0.99), assessment (AOR 0.84, 95% CI 0.75-0.96), refraining from specific behaviors (AOR 0.78, 95% CI 0.70-0.88), economic and social effects (AOR 0.83, 95% CI 0.72-0.94), and dealing with psychological stress (AOR 0.77, 95% CI 0.66-0.90).

**Table 8:** The proportion of “yes” responses to questions on searching each topic about COVID-19.

Topic about COVID-19	Participants (N=6000), n (%)
The infectivity of the novel coronavirus	4015 (66.92)
Information about the novel coronavirus vaccine	3650 (60.83)
Symptoms of the novel coronavirus	2494 (41.57)
Things individuals can do to prevent novel coronavirus infection	1920 (32)
Refraining from certain behaviors	1915 (31.92)
Rules and behavior regarding novel coronavirus infection prevention	1530 (25.5)
Assessment of the current novel coronavirus infection status and recommendations	1445 (24.08)
The economic and social effects of the novel coronavirus	1260 (21)
Route of infection of the novel coronavirus	1172 (19.53)
How to deal with the psychological stress caused by the novel coronavirus	775 (12.92)

**Table 9:** Associations of eHL^a^ levels with searching specific COVID-19 topics.

eHL		The infectivity		Route of infection		Symptoms		Preventive measures		Rules and behaviors	
		Value, n (%)	AOR^b^ (95% CI)^c^	Value, n (%)	AOR (95% CI)^c^	Value, n (%)	AOR (95% CI)^c^	Value, n (%)	AOR (95% CI)^c^	Value, n (%)	AOR (95% CI)^c^
**eHEALS^d^**											
	Low (n=2228)	1401 (62.88)	1.00 (reference)	319 (14.32)	1.00 (reference)	755 (33.89)	1.00 (reference)	553 (24.82)	1.00 (reference)	400 (17.95)	1.00 (reference)
	High (n=3772)	2614 (69.3)	1.25 (1.11-1.40)	853 (22.61)	1.63 (1.41-1.89)	1739 (46.1)	1.58 (1.41-1.77)	1367 (36.24)	1.58 (1.40-1.79)	1130 (29.96)	1.78 (1.56-2.03)
**Information search**											
	Low (n=1613)	1010 (62.62)	1.00 (reference)	273 (16.92)	1.00 (reference)	646 (40.05)	1.00 (reference)	491 (30.44)	1.00 (reference)	388 (24.05)	1.00 (reference)
	High (n=4387)	3005 (68.5)	1.25 (1.11-1.41)	899 (20.49)	1.21 (1.04-1.41)	1848 (42.12)	1.06 (0.94-1.19)	1429 (32.57)	1.07 (0.94-1.22)	1142 (26.03)	1.08 (0.94-1.24)
**Adding self-generated information**											
	Low (n=2702)	1771 (65.54)	1.00 (reference)	453 (16.77)	1.00 (reference)	1124 (41.6)	1.00 (reference)	824 (30.5)	1.00 (reference)	653 (24.17)	1.00 (reference)
	High (n=3298)	2244 (68.04)	1.05 (0.94-1.18)	719 (21.8)	1.29 (1.12-1.47)	1370 (41.54)	0.96 (0.86-1.07)	1096 (33.23)	1.09 (0.98-1.23)	877 (26.59)	1.09 (0.97-1.23)
**Evaluating reliability**											
	Low (n=2475)	1655 (66.87)	1.00 (reference)	403 (16.28)	1.00 (reference)	1022 (41.29)	1.00 (reference)	772 (31.19)	1.00 (reference)	611 (24.69)	1.00 (reference)
	High (n=3525)	2360 (66.95)	0.96 (0.86-1.08)	769 (21.82)	1.33 (1.16-1.53)	1472 (41.76)	1.02 (0.92-1.14)	1148 (32.57)	1.09 (0.97-1.22)	919 (26.07)	1.10 (0.97-1.24)
**Determining relevance**											
	Low (n=2051)	1350 (65.82)	1.00 (reference)	344 (16.77)	1.00 (reference)	820 (39.98)	1.00 (reference)	594 (28.96)	1.00 (reference)	473 (23.06)	1.00 (reference)
	High (n=3949)	2665 (67.49)	1.03 (0.92-1.16)	828 (20.97)	1.23 (1.07-1.42)	1674 (42.39)	1.05 (0.94-1.18)	1326 (33.58)	1.18 (1.05-1.34)	1057 (26.77)	1.16 (1.02-1.32)
**Operational skills**											
	Low (n=1076)	625 (58.09)	1.00 (reference)	187 (17.38)	1.00 (reference)	385 (35.78)	1.00 (reference)	284 (26.39)	1.00 (reference)	234 (21.75)	1.00 (reference)
	High (n=4924)	3390 (68.85)	1.59 (1.38-1.83)	985 (20)	1.10 (0.92-1.31)	2109 (42.83)	1.37 (1.19-1.58)	1636 (33.23)	1.48 (1.27-1.73)	1296 (26.32)	1.39 (1.18-1.64)
**Navigation skills**											
	Low (n=1905)	1273 (66.82)	1.00 (reference)	433 (22.73)	1.00 (reference)	822 (43.15)	1.00 (reference)	639 (33.54)	1.00 (reference)	506 (26.56)	1.00 (reference)
	High (n=4095)	2742 (66.96)	1.06 (0.94-1.19)	739 (18.05)	0.77 (0.67-0.89)	1672 (40.83)	0.96 (0.86-1.08)	1281 (31.28)	0.98 (0.86-1.10)	1024 (25.01)	1.01 (0.89-1.16)
**Privacy protection**											
	Low (n=2206)	1490 (67.54)	1.00 (reference)	515 (23.35)	1.00 (reference)	1002 (45.42)	1.00 (reference)	816 (36.99)	1.00 (reference)	620 (28.11)	1.00 (reference)
	High (n=3794)	2525 (66.55)	1.02 (0.91-1.15)	657 (17.32)	0.71 (0.63-0.82)	1492 (39.33)	0.81 (0.73-0.90)	1104 (29.1)	0.74 (0.66-0.83)	910 (23.99)	0.87 (0.77-0.99)
**Total DHLI** ^e^ **score**											
	Low (n=3264)	2142 (65.63)	1.00 (reference)	614 (18.81)	1.00 (reference)	1378 (42.22)	1.00 (reference)	1053 (32.26)	1.00 (reference)	833 (25.52)	1.00 (reference)
	High (n=2736)	1873 (68.46)	1.14 (1.02-1.28)	558 (20.39)	1.06 (0.93-1.21)	1116 (40.79)	0.95 (0.86-1.06)	867 (31.69)	1.01 (0.90-1.13)	697 (25.48)	1.04 (0.92-1.18)

^a^eHL: eHealth literacy.

^b^AOR: adjusted odds ratio.

^c^The multivariable logistic regression model that adjusted for all covariates (ie, gender, age groups, equivalent income, education status, marital status, cigarette smoking, alcohol consumption, physical exercise habit, and conditions leading to severe illness due to COVID-19).

^d^eHEALS: eHealth Literacy Scale.

^e^DHLI: Digital Health Literacy Instrument.

**Table 10:** Associations of eHL^a^ levels with searching the specific COVID-19 topics (continued).

eHL		Assessment of the current novel coronavirus infection status			Refraining from specific behaviors			The economic and social effects of the novel coronavirus			Dealing with the psychological stress caused by the novel coronavirus			Information about the novel coronavirus vaccine	
		Value, n (%)	AOR^b^ (95% CI)^c^	Value, n (%)		AOR (95% CI)^c^	Value, n (%)		AOR (95% CI)^c^	Value, n (%)		AOR (95% CI)^c^	Value, n (%)		AOR (95% CI)^c^
**eHEALS^d^**															
	Low (n=2228)	371 (16.65)	1.00 (reference)	579 (25.99)		1.00 (reference)	322 (14.45)		1.00 (reference)	153 (6.87)		1.00 (reference)	1229 (55.16)		1.00 (reference)
	High (n=3772)	1074 (28.47)	1.81 (1.58-2.07)	1336 (35.42)		1.43 (1.27-1.61)	938 (24.87)		1.83 (1.59-2.11)	622 (16.49)		2.47 (2.04-2.98)	2421 (64.18)		1.43 (1.28-1.60)
**Information search**															
	Low (n=1613)	333 (20.64)	1.00 (reference)	498 (30.87)		1.00 (reference)	299 (18.54)		1.00 (reference)	167 (10.35)		1.00 (reference)	956 (59.27)		1.00 (reference)
	High (n=4387)	1112 (25.35)	1.24 (1.08-1.43)	1417 (32.3)		1.02 (0.90-1.16)	961 (21.91)		1.19 (1.02-1.38)	608 (13.86)		1.35 (1.12-1.63)	2694 (61.41)		1.13 (1.01-1.28)
**Adding self-generated information**															
	Low (n=2702)	614 (22.72)	1.00 (reference)	859 (31.79)		1.00 (reference)	514 (19.02)		1.00 (reference)	306 (11.32)		1.00 (reference)	1692 (62.62)		1.00 (reference)
	High (n=3298)	831 (25.2)	1.05 (0.92-1.19)	1056 (32.02)		0.94 (0.84-1.06)	746 (22.62)		1.18 (1.04-1.34)	469 (14.22)		1.23 (1.05-1.44)	1958 (59.37)		0.91 (0.81-1.01)
**Evaluating reliability**															
	Low (n=2475)	544 (21.98)	1.00 (reference)	789 (31.88)		1.00 (reference)	462 (18.67)		1.00 (reference)	290 (11.72)		1.00 (reference)	1536 (62.06)		1.00 (reference)
	High (n=3525)	901 (25.56)	1.17 (1.03-1.32)	1126 (31.94)		0.99 (0.89-1.12)	798 (22.64)		1.24 (1.09-1.42)	485 (13.76)		1.17 (1.00-1.38)	2114 (59.97)		1.00 (0.90-1.12)
**Determining relevance**															
	Low (n=2051)	429 (20.92)	1.00 (reference)	615 (29.99)		1.00 (reference)	391 (19.06)		1.00 (reference)	230 (11.21)		1.00 (reference)	1222 (59.58)		1.00 (reference)
	High (n=3949)	1016 (25.73)	1.22 (1.07-1.40)	1300 (32.92)		1.09 (0.97-1.23)	869 (22.01)		1.14 (1.00-1.31)	545 (13.8)		1.19 (1.01-1.41)	2428 (61.48)		1.10 (0.98-1.23)
**Operational skills**															
	Low (n=1076)	177 (16.45)	1.00 (reference)	292 (27.14)		1.00 (reference)	164 (15.24)		1.00 (reference)	127 (11.8)		1.00 (reference)	582 (54.09)		1.00 (reference)
	High (n=4924)	1268 (25.75)	1.73 (1.44-2.07)	1623 (32.96)		1.34 (1.15-1.57)	1096 (22.26)		1.57 (1.30-1.88)	648 (13.16)		1.15 (0.93-1.42)	3068 (62.31)		1.60 (1.39-1.84)
**Navigation skills**															
	Low (n=1905)	454 (23.83)	1.00 (reference)	661 (34.7)		1.00 (reference)	421 (22.1)		1.00 (reference)	277 (14.54)		1.00 (reference)	1093 (57.38)		1.00 (reference)
	High (n=4095)	991 (24.2)	1.07 (0.94-1.22)	1254 (30.62)		0.88 (0.78-0.99)	839 (20.49)		0.94 (0.82-1.08)	498 (12.16)		0.86 (0.73-1.01)	2557 (62.44)		1.30 (1.16-1.45)
**Privacy protection**															
	Low (n=2206)	589 (26.7)	1.00 (reference)	799 (36.22)		1.00 (reference)	521 (23.62)		1.00 (reference)	334 (15.14)		1.00 (reference)	1305 (59.16)		1.00 (reference)
	High (n=3794)	856 (22.56)	0.84 (0.75-0.96)	1116 (29.41)		0.78 (0.70-0.88)	739 (19.48)		0.83 (0.72-0.94)	441 (11.62)		0.77 (0.66-0.90)	2345 (61.81)		1.16 (1.04-1.29)
**Total DHLI^e^ score**															
	Low (n=3264)	751 (23.01)	1.00 (reference)	1054 (32.29)		1.00 (reference)	645 (19.76)		1.00 (reference)	404 (12.38)		1.00 (reference)	1980 (60.66)		1.00 (reference)
	High (n=2736)	694 (25.37)	1.12 (0.99-1.27)	861 (31.47)		0.98 (0.87-1.09)	615 (22.48)		1.18 (1.03-1.34)	371 (13.56)		1.11 (0.95-1.29)	1670 (61.04)		1.10 (0.99-1.23)

^a^eHL: eHealth literacy.

^b^AOR: adjusted odds ratio.

^c^The multivariable logistic regression model that adjusted for all covariates (gender, age groups, equivalent income, education status, marital status, cigarette smoking, alcohol consumption, physical exercise habit, and conditions leading to severe illness due to COVID-19).

^d^eHEALS: eHealth Literacy Scale.

^e^DHLI: Digital Health Literacy Instrument.

### Difficulties in Seeking and Using COVID-19 Information

Difficulties in seeking and using COVID-19 information were examined using a qualitative content analysis of 6000 valid answers to open-ended questions. Excluding 3151 (52.52%) participants who responded as perceiving no difficulties, we have listed the top 50 categories and themes (Table 11). “Information quality and credibility,” as theme I, included *information discernment* and *disinformation.* “Abundance and shortage of relevant information,” as theme II, included *incomprehensible information* and *information overload*. “Public trust and skepticism,” as theme III, included *doubting (local) governments* and *doubting specialists and doctors*. “Credibility of COVID-19–related information,” as theme IV, included *vaccination information*. These themes, including top 10 categories, cover common difficulties among people. “Privacy and security concerns,” as theme V, included *protecting personal information*. “Information retrieval challenges,” as theme VI, included *time-consuming information search*. “Anxieties and panic,” as theme VII, included *anxiety and panic*. “Movement restriction,” as theme VIII, included *time-consuming information search*. The number of categories in themes V to VIII was fewer than that in themes I to IV, indicating that the latter themes were related with relatively more specific difficulties.

Moreover, we analyzed the association between eHL and difficulties in seeking and using COVID-19 information (Table 12). The participants with higher total DHLI scores were more likely not to respond and be disinformed and less likely to answer questions on *information discernment*, *incomprehensible information*, and *information overload*. Half of the participants (3151/6000, 52.52%) reported no difficulty seeking and using COVID-19 information. Participants reporting *no* difficulties (*P* for trend=.01) and *incomprehensible information* (*P*<.001) demonstrated lower eHEALS scores. Regarding the DHLI, participants reporting *no* difficulties (*P*<.001) demonstrated a higher total DHLI score, while those reporting *information discernment* (*P*<.001), *incomprehensible information* (*P*<.001), *information overload* (*P*=.003), and *disinformation* (*P*=.02) had a lower score.

**Table 11:** Top 50 categories and themes of difficulties in seeking and using COVID-19 information.

Categories	Themes								
	I^a^	II^b^	III^c^	IV^d^	V^e^	VI^f^	VII^g^	VIII^h^	IX^i^
1. Information discernment	✓								
2. Incomprehensible information		✓							
3. Information overload		✓							
4. Vaccination information				✓					
5. Disinformation	✓								
6. Lack of information meeting their needs		✓							
7. Information without evidence	✓								
8. Information without credibility or trust	✓								
9. Lack of detailed patient information		✓							
10. Doubting (local) governments			✓						
11. Lack of information concerning their local area		✓							
12. Not seeking information									✓
13. Conflicting information	✓								
14. Lack of up-to-date information		✓							
15. Anxiety and panic						`	✓		
16. Rabble-rousing information	✓								
17. Insufficient aggregated information of patients		✓							
18. Doubting specialists and doctors			✓						
19. Doubting the media			✓						
20. Lack of information after infection		✓							
21. Misinformation	✓								
22. Information control and manipulation	✓								
23. Information resources	✓								
24. Time-consuming information search						✓			
25. Lack of information on prospects		✓							
26. Technical terms and jargon		✓							
27. No answers to unknown virus				✓					
28. Lack of information about other countries		✓							
29. Redundant or repetitive information		✓							
30. Information on infection risk and prevention				✓					
31. Lack of information on COVID-19 testing				✓					
32. Doubting the social media			✓						
33. Lack of information on the availability of essential services		✓							
34. Antivaccination and antigovernment	✓								
35. Lack of comprehensive information		✓							
36. Regulation and self-restraint								✓	
37. Operating PCs and smartphones						✓			
38. Protecting personal information					✓				
39. Lack of high-quality information		✓							
40. The early stage of COVID-19				✓					
41. Doubting various authorities that lack cooperation			✓						
42. How to deal with information	✓								
43. Information on advertisement		✓							
44. Information on SARS-CoV-2				✓					
45. Differentiating COVID-19 from a cold				✓					
46. Lack of information suitable for oneself		✓							
47. Imbalance in information toward metropolitan areas		✓							
48. Lack of information for close contacts		✓							
49. Financial hardship									✓
50. Trust in authorities			✓						

^a^Theme I: information quality and credibility.

^b^Theme II: abundance and shortage of relevant information.

^c^Theme III: public trust and skepticism.

^d^Theme IV: credibility of COVID-19–related information.

^e^Theme V: privacy and security concerns.

^f^Theme VI: information retrieval challenges.

^g^Theme VII: anxieties and panic.

^h^Theme VIII: movement restriction.

^i^Theme IX: others.

**Table 12:** Associations of eHL^a^ with difficulties in seeking and using COVID-19 information (none and the top 5 difficulties).

Difficulties	Total (n=6000), n (%)	eHEALS^b^ (quartile)					*P* for trend		Total score of DHLI^c^ (quartile)					*P* for trend
		Q1 (Low) (n=1504), n (%)	Q2 (n=1531), n (%)	Q3 (n=1429), n (%)	Q4 (High) (n=1536), n (%)			Q1 (Low) (n=1437), n (%)		Q2 (n=1511), n (%)	Q3 (n=1531), n (%)	Q4 (High) (n=1521), n (%)		
None	3151 (52.52)	779 (51.8)	899 (58.72)	707 (49.48)	766 (49.87)	.01		628 (43.7)		738 (48.84)	845 (55.19)	940 (61.8)	<.001	
Information discernment	409 (6.82)	103 (6.85)	94 (6.14)	115 (8.05)	97 (6.32)	.94		116 (8.07)		135 (8.93)	86 (5.62)	72 (4.73)	<.001	
Incomprehensible information	348 (5.8)	140 (9.31)	87 (5.68)	66 (4.62)	55 (3.58)	<.001		139 (9.67)		95 (6.29)	67 (4.38)	47 (3.09)	<.001	
Information overload	272 (4.53)	64 (4.26)	69 (4.51)	70 (4.9)	69 (4.49)	.65		68 (4.73)		89 (5.89)	73 (4.77)	42 (2.76)	.003	
Vaccination information	261 (4.35)	60 (3.99)	54 (3.53)	75 (5.25)	72 (4.69)	.11		61 (4.24)		73 (4.83)	70 (4.57)	57 (3.75)	.45	
Disinformation	209 (3.48)	44 (2.93)	56 (3.66)	51 (3.57)	58 (3.78)	.24		41 (2.85)		47 (3.11)	55 (3.59)	66 (4.34)	.02	

^a^eHL: eHealth literacy.

^b^eHEALS: eHealth Literacy Scale.

^c^DHLI: Digital Health Literacy Instrument.

## Discussion

### Principal Findings

This study using mixed methods is the first to examine the associations between eHL and web-based health information–seeking behaviors and to identify the difficulties in using health information on the internet and the its relationship with eHL among adult internet users. Internet users with high eHEALS and DHLI scores on information searching, adding self-generated content, evaluating reliability, determining relevance, and operational skills were more likely to use all web sources of information about COVID-19 than those with low eHEALS and DHLI scores. However, there were negative associations between navigation skill scores and privacy protection when using several information sources, such as YouTube, to search for COVID-19 information. In addition, participants with high eHEALS and DHLI scores on information searching, adding self-generated information, evaluating reliability, and determining relevance were more likely to search for information about COVID-19 than those with low eHEALS or DHLI scores. However, some participants with high navigation skills and privacy protection skills were less likely to search for information on COVID-19. Furthermore, this study shed light on the difficulties seeking and using COVID-19 information qualitatively. While half of the participants reported no difficulty seeking and using COVID-19 information, participants who reported any difficulties, including *information discernment*, *incomprehensible information*, *information overload*, and *disinformation*, had lower DHLI score. Finally, participants expressed significant concerns regarding “information quality and credibility,” “abundance and shortage of relevant information,” “public trust and skepticism,” and “credibility of COVID-19–related information.” In addition, they disclosed more specific concerns, including “privacy and security concerns,” “information retrieval challenges,” “anxieties and panic,” and “movement restriction.”

The study results suggest that internet users with higher eHEALS and total DHLI scores were more likely to use a reliable information source, consistent with prior studies [14,20,31]. Considering the subscale of DHLI, the ability to determine relevance of and evaluate the reliability of information is reportedly positively associated with the search for COVID-19 information through a traditional 1-way communication channel known as “Health 1.0,” involving public institution websites [15-18,22,32]. In addition, a previous study showed positive associations of higher skills in information searching and adding self-generated content with using public institution websites [15]. However, to our knowledge, no study has examined the association between operational skills and web-based health information–seeking behavior because most studies have focused on university students and have not assessed operational information skills [14-18]. Operational skills, which are basic skills required to use computers, are vital for searching web-based health information, with implications among people unfamiliar with computers or smartphones, such as older adults. A Japanese government survey indicated that deficiencies in the basic skills required to use computers or smartphones were barriers to internet access among older adults [1]. Therefore, this finding suggests that operational skills are critical for using web-based health information among individuals who are generally less familiar with the internet.

There were negative associations between navigation and protecting privacy skills and using several web-based interactive health-related communications channels via SNSs, such as YouTube. Several studies have reported that university students with low DHL scores are more likely to use Health 2.0 channels for health information than university students with high DHLI scores [14,17]. In addition, the study findings showed that participants with high navigation and protecting privacy skills were less likely to search for information on, for example, the route of infection or on refraining from certain behaviors. Participants with high navigation and protecting privacy skills used web-based information about COVID-19 cautiously compared to those with lower navigation and proficiency skills. However, there was no negative association between eHEALS scores and the use of Health 2.0 communication channels. This result could be explained by the reason that eHEALS scores did not encompass the skills required to use Health 2.0 [33]. The eHEALS would need to be improved for adaptation to Health 2.0 communication channels.

The leading 50 categories related to difficulties seeking and using COVID-19 information were identified using a qualitative approach. Our findings indicate that approximately half of the participants experienced difficulties. Information discernment was the most common issue. Health literacy encompasses functional, interactive, and critical literacies [34]. Information discernment is a crucial aspect of literacy. It concerns an individual’s ability to discriminate misinformation from accurate information. It has been assessed by calculating the difference in scores related to discerning accurate information from misinformation [35,36]. Managing the volume of available information and assessing its quality and reliability are essential DHL skills [37]. Our results revealed that information discernment was not linked to proficiency in terms of eHEALS scores but rather to DHLI scores. This finding underscores a pivotal shift in the Health 2.0 era when basic knowledge of Health 1.0 health literacy is insufficient. Our study highlights the need for an enhanced level of health literacy tailored to facilitate the navigation of the complexities and nuances of information in the Health 2.0 landscape.

We qualitatively presented themes related to difficulties in seeking and using COVID-19 information. Themes should be evaluated against the backdrop of previous studies. Several tools or instruments for assessing the quality of health information have been used extensively, such as the *Journal of the American Medical Association* benchmarks, Sandvik’s general quality criteria, DISCERN, HONcode by the Health on the Net Foundation, and quality evaluation scoring tools [38]. Denniss et al [39] recently developed the 13 Principles for Health-Related Information on Social Media (PRHISM). The US National Academy of Medicine has proposed 3 foundational principles to guide the identification of credible sources of health information on social media, namely, that they are science based, objective, transparent, and accountable [40]. “Information quality and credibility” and “credibility of COVID-19–related information” were difficulties identified in relation to science-based principles, and “relevant information” included science-based information. “Skepticism” could arise from lacking objectives, transparency, and accountability. These aspects generally align with the PRHISM. In addition, our findings highlight the significance of people-centered or narrative information. Relevant information includes detailed patient information, information suitable for patients, and information concerning the local area. Simultaneously, the demand for narrative information could lead to “privacy and security concerns,” emphasizing the need to balance this aspect with personal information protection. The categories and themes could be used to develop a comprehensive list of challenges likely to be faced during future infodemics.

It is widely acknowledged that the COVID-19 pandemic has brought to light the impact of disparities on health outcomes, thereby highlighting the imperative to tackle these inequalities. Alongside health literacy and other social determinants of health [34], the pandemic has underscored the significance of information as an independent determinant of health [41]. Limited access to high-quality information can exacerbate disparities, particularly in education and economic stability. Addressing such disparities requires collaborative efforts among stakeholders worldwide. Establishing meaningful partnerships between governmental, nongovernmental, and private-sector organizations is crucial to the creation of governance frameworks to counter information-related threats. Given the ongoing pivotal role of information in shaping health outcomes, collective action is essential to mitigate the adverse impact of misinformation. To achieve this, a fundamental framework is needed to universally enhance DHL and improve web-based content. Our insights will provide a valuable contribution to the creation of such a framework.

### Limitations

This study has some limitations. First, participants were recruited from a single Japanese internet research service company. These participants were suitable for an internet-based survey because internet users need to have adequate eHL, and the participants were equally divided by gender, age, and income and then recruited by a research company. However, the results may have been biased, as our participants could have a disproportionately higher educational status and higher eHL skill levels than general internet users [42-44]. Therefore, the eHEALS scores, DHLI scores, and proportion of those searching for COVID-19 information identified in this study may have been higher than those among general internet users in Japan. Second, the study findings may not be directly generalizable to other countries because of inherent differences in website and SNS environments. Third, although previous studies have objectively evaluated the eHL of participants using performance tests [22,45], this study did not objectively assess the eHL and DHLI dimensions. Therefore, there may have been inaccuracies in estimating participants’ eHL levels. Finally, this study’s cross-sectional design means that causality remains unknown.

### Conclusions

This study revealed that Japanese individuals with higher eHEALS and DHLI scores were more engaged in using various web sources when seeking COVID-19 information. However, proficiency in terms of the eHEALS may not encompass the skills required to use Health 2.0. Higher scores on navigation and privacy protection skills, not included in the eHEALS, correlated with less use of specific sources, such as YouTube. Participants with high navigation and privacy protection skills used web-based information about COVID-19 cautiously compared with those with lower proficiency in these skills. This study also highlights an increased need for information discernment in the Health 2.0 era. The identified categories and themes, such as “information quality and credibility,” “abundance and shortage of relevant information,” “public trust and skepticism,” and “privacy and security concerns,” suggest a framework that could be used to address the myriad challenges anticipated in future infodemics. In the future, we aim to compile a comprehensive list of such challenges. To strengthen the public’s resilience against misinformation, a fundamental framework should be established to enhance DHL for all individuals and improve the quality of web-based content.

## Appendix

Multimedia Appendix 1 Digital Health Literacy Instrument adapted to the COVID-19 pandemic and web-based information–seeking behavior on COVID-19 in Japanese.

## Abbreviations

AOR: adjusted odds ratio
COVID-HL: COVID-19 health literacy
DHL: digital health literacy
DHLI: Digital Health Literacy Instrument
eHEALS: eHealth Literacy Scale
eHL: eHealth literacy
J-eHEALS: Japanese version of the eHealth Literacy Scale
PRHISM: 13 Principles for Health-Related Information on Social Media
SNS: social networking site
