# Supplementary material for: eHealth Literacy and Web-Based Health Information–Seeking Behaviors on COVID-19 in Japan: Internet-Based Mixed Methods Study
Source: J Med Internet Res. 2024 Jul 11;26:e57842. doi: 10.2196/57842 (PMC11273073; doi:10.2196/57842)
Supplement: Multimedia Appendix 1 [file jmir_v26i1e57842_app1.docx]

Supplemental table 1. Digital Health Literacy Instrument adapted to the COVID-19 and online information-seeking behavior on the COVID-19 in Japanese

| Digital Health literacy Instrument adapted to the COVID-19 in Japanese |
| --- |

| 1. | あなたは、下記の内容について、どの程度難しく感じますか。 | | | | |
| --- | --- | --- | --- | --- | --- |
|  |  | とても　簡単 | 簡単 | 難しい | とても　難しい |
|  | コンピューターのキーボードを使うこと（例：言葉の入力） |  |  |  |  |
|  | マウスを使うこと（例：カーソルの移動やクリック） |  |  |  |  |
|  | ウェブサイトで、ボタンやリンク、ハイパーリンクを使うこと |  |  |  |  |

| 2. | あなたが新型コロナウイルス感染症に関連する情報を探す時、どのくらいの頻度で下記のことが生じますか。 | | | | |
| --- | --- | --- | --- | --- | --- |
|  |  | ない | 一度 | 数回 | しばしば |
|  | ウェブサイト内やインターネットで、どこを探していたかわからなくなる |  |  |  |  |
|  | 前のページに戻る方法がわからなくなる |  |  |  |  |
|  | クリックした際、思っていたこととは違うことが生じる |  |  |  |  |

| 3. | 新型コロナウイルス感染症に関連する情報をインターネットで探す場合、下記の内容について、あなたはどの程度の難しさを感じますか。 | | | | |
| --- | --- | --- | --- | --- | --- |
|  |  | とても　簡単 | 簡単 | 難しい | とても　難しい |
|  | あなたが見つけた情報を取捨選択すること |  |  |  |  |
|  | あなたが探している情報を見つけるために、適切な検索ワードや検索式を使うこと |  |  |  |  |
|  | あなたが探している情報と合致するものを見つけること |  |  |  |  |

| 4. | 新型コロナウイルス感染症に関連するメッセージ（例：ウェブフォーラムやFacebookやTwitterなどのソーシャルメディア）を投稿するとしたら、下記の内容について、あなたはどの程度の難しさを感じますか。 | | | | |
| --- | --- | --- | --- | --- | --- |
|  |  | とても　簡単 | 簡単 | 難しい | とても　難しい |
|  | あなたの質問や健康に関する心配ごとを明確に書くこと |  |  |  |  |
|  | あなたの意見や考え、感情を表現すること |  |  |  |  |
|  | あなたが意図するメッセージの意味を、他の人が正確に理解できるように書くこと |  |  |  |  |

| 5. | 新型コロナウイルス感染症に関連する情報をインターネットで探す場合、下記の内容について、あなたはどの程度の難しさを感じますか。 | | | | |
| --- | --- | --- | --- | --- | --- |
|  |  | とても　簡単 | 簡単 | 難しい | とても　難しい |
|  | 情報が信頼できるかどうかを判断すること |  |  |  |  |
|  | 情報が商業目的（例：製品の販売）で書かれているかどうかを判断すること |  |  |  |  |
|  | 同じ情報が提供されているかどうかを他のウェブサイトで確認すること |  |  |  |  |

| 6. | 新型コロナウイルス感染症に関連する情報をインターネットで探す場合、下記の内容について、あなたはどの程度の難しさを感じますか。 | | | | |
| --- | --- | --- | --- | --- | --- |
|  |  | とても　簡単 | 簡単 | 難しい | とても　難しい |
|  | あなたが見つけた情報が、あなた自身にあてはまる情報であったかどうかを判断すること |  |  |  |  |
|  | あなたが見つけた情報を、日常生活で活用すること |  |  |  |  |
|  | あなたが見つけた情報を、あなた自身の健康に関する意思決定に使うこと（例：行動の自粛や予防行動） |  |  |  |  |

| 7. | 新型コロナウイルス感染症に関連するメッセージをウェブフォーラムやソーシャルメディア（FacebookやTwitterなど）に投稿するとしたら、どのくらいの頻度で下記のことがありますか。 | | | | |
| --- | --- | --- | --- | --- | --- |
|  |  | ない | 一度 | 数回 | しばしば |
|  | あなたが投稿したメッセージを、誰が読むことができるか知ることは難しいと感じること |  |  |  |  |
|  | あなた自身の個人情報（例：名前や住所）が共有されること（意識的にでも、無意識的にでも） |  |  |  |  |
|  | 他人の個人情報を共有すること（意識的にでも、無意識的にでも） |  |  |  |  |

| Online information-seeking behavior on the COVID-19 in Japanese |
| --- |

| 8. | インターネット上で、新型コロナウイルス感染症に関連する情報を収集するには、さまざまな情報源があります。下記の情報源を最近どのくらい使いましたか。 | | | | | |
| --- | --- | --- | --- | --- | --- | --- |
|  |  | しばしば | 時々 | めったにない | まったくない | わからない |
|  | 検索エンジン（例：Google、Bing、Yahoo!） |  |  |  |  |  |
|  | 公共機関によるウェブサイト（例：厚生労働省、医師会） |  |  |  |  |  |
|  | ウィキペディアやウェブベースの百科事典 |  |  |  |  |  |
|  | ソーシャルメディア （例：Facebook、Instagram、Twitter） |  |  |  |  |  |
|  | YouTube |  |  |  |  |  |
|  | 医療健康情報に関するブログ |  |  |  |  |  |
|  | 医療健康に関連したQ&Aサイト（例：Yahoo!知恵袋） |  |  |  |  |  |
|  | 医療健康情報ポータルサイト |  |  |  |  |  |
|  | 医師や医療機関が運営するウェブサイト |  |  |  |  |  |
|  | ニュースポータルサイト（例：新聞社やテレビ局からの情報） |  |  |  |  |  |

| 9. | 新型コロナウイルス感染症について、どのような情報を具体的に検索しているかお答えください。（複数回答可） | |
| --- | --- | --- |
|  | □ | 新型コロナウイルスの感染状況（例：感染者数） |
|  | □ | 新型コロナウイルスの感染経路 |
|  | □ | 新型コロナウイルスにかかった場合の症状 |
|  | □ | 感染予防のためにできる個人の対策（例：手洗い） |
|  | □ | 感染予防のための規制やマナー（例：消毒や洗浄） |
|  | □ | 現在の感染状況の評価（宣言・措置・ステージなど）と推奨事項（例：厚生労働省や都道府県からの情報） |
|  | □ | 行動の自粛（例：出国・都道府県をまたいだ移動・外出の自粛） |
|  | □ | 新型コロナウイルスによる経済的および社会的影響 |
|  | □ | 新型コロナウイルスによる心理的ストレスへの対処方法 |
|  | □ | ワクチンに関する情報（例：有効性や副反応、接種状況） |
